# Sternoclavicular joint infection caused by *Coxiella burnetii*: a case report

**DOI:** 10.1186/s13256-016-0948-x

**Published:** 2016-05-31

**Authors:** Emmanouil Angelakis, Simon-Djamel Thiberville, Matthieu Million, Didier Raoult

**Affiliations:** 1 URMITE, UM63, CNRS 7278, IRD 198, Inserm 1095, Aix-Marseille Université, 13005 Marseille, France; 2 UMR190, Aix-Marseille Université / IRD / EHESP French School of Public Health, Marseille, France; 3 Hospital Louis Raffalli, Medicine D Unit, Infectious Disease and Internal Medicine, Manosque, France

**Keywords:** *Coxiella burnetii*, Q fever, Osteoarticular infection, Case report, Sternoclavicular joint infection

## Abstract

**Background:**

Few cases of Q fever osteoarticular infection have been reported, with chronic osteomyelitis as the most common manifestation of Q fever osteoarticular infection. Here we present the case of a sternoclavicular joint infection caused by *Coxiella burnetii* and localized by positron emission tomography scanning.

**Case presentation:**

A 67-year-old French man from south France was hospitalized for fever and confusion. An examination revealed subclavicular and axillary lymph node enlargement. Computed tomography scanning and transesophageal echocardiogram were normal, and magnetic resonance imaging scanning did not reveal signs of infection. An immunofluorescence assay of an acute serum sample was positive for *C. burnetii* and he was treated with 200 mg doxycycline for 21 days. An immunofluorescence assay of convalescent serum sampled after 2 months revealed very high *C. burnetii* antibody titers. To localize the site of the infection, we performed positron emission tomography scanning, which revealed intense fluorodeoxyglucose uptake in his right sternoclavicular joint; treatment with 200 mg oral doxycycline daily and 200 mg oral hydroxychloroquine three times daily for 18 months was initiated.

**Conclusions:**

Q fever articular infections may be undiagnosed, and we strongly urge the use of positron emission tomography scanning in patients with high *C. burnetii* antibody titers to localize the site of *C. burnetii* infection.

## Background

Q fever is a potentially life-threatening worldwide zoonosis caused by an obligate intracellular bacterium, *Coxiella burnetii* [1]. Infective endocarditis is the most frequent Q fever chronic infection, followed by vascular, osteoarticular, hepatitis and pulmonary infection [1]. To date, few cases of Q fever osteoarticular infection have been reported in the literature; they include osteomyelitis, spondylodiscitis and two cases of tenosynovitis [2, 3]. In fact, only seven (2 %) osteoarticular infections were detected in a large serologic study which included more than 1300 cases of Q fever that extended over 14 years [4], and 11 (0.7 %) cases in a recent 7-year study which included more than 1400 cases [5]. Osteomyelitis is the most common manifestation of Q fever osteoarticular infection, followed by vertebral spondylodiscitis and paravertebral abscess [1, 2]. *C. burnetii* has also been implicated in a prosthetic joint infection [3], while two cases of tenosynovitis have been reported [6].

Q fever osteoarticular infection can easily go undiagnosed because of the long evolution of articular involvement, which is accompanied by a low level of laboratory and inflammatory signs [1]. However, in recent years, positron emission tomography (PET) scanning has been successfully used for the identification of infectious foci in *C. burnetii* infections [1, 7], and the use of PET scanning was recently proposed as a complementary tool for patients with high *C. burnetii* antibody titers in order to localize the site of *C. burnetii* infection [1, 8]. Here we present a case of a sternoclavicular joint infection caused by *C. burnetii*, localized by PET scanning.

## Case presentation

**Fig. 1:**
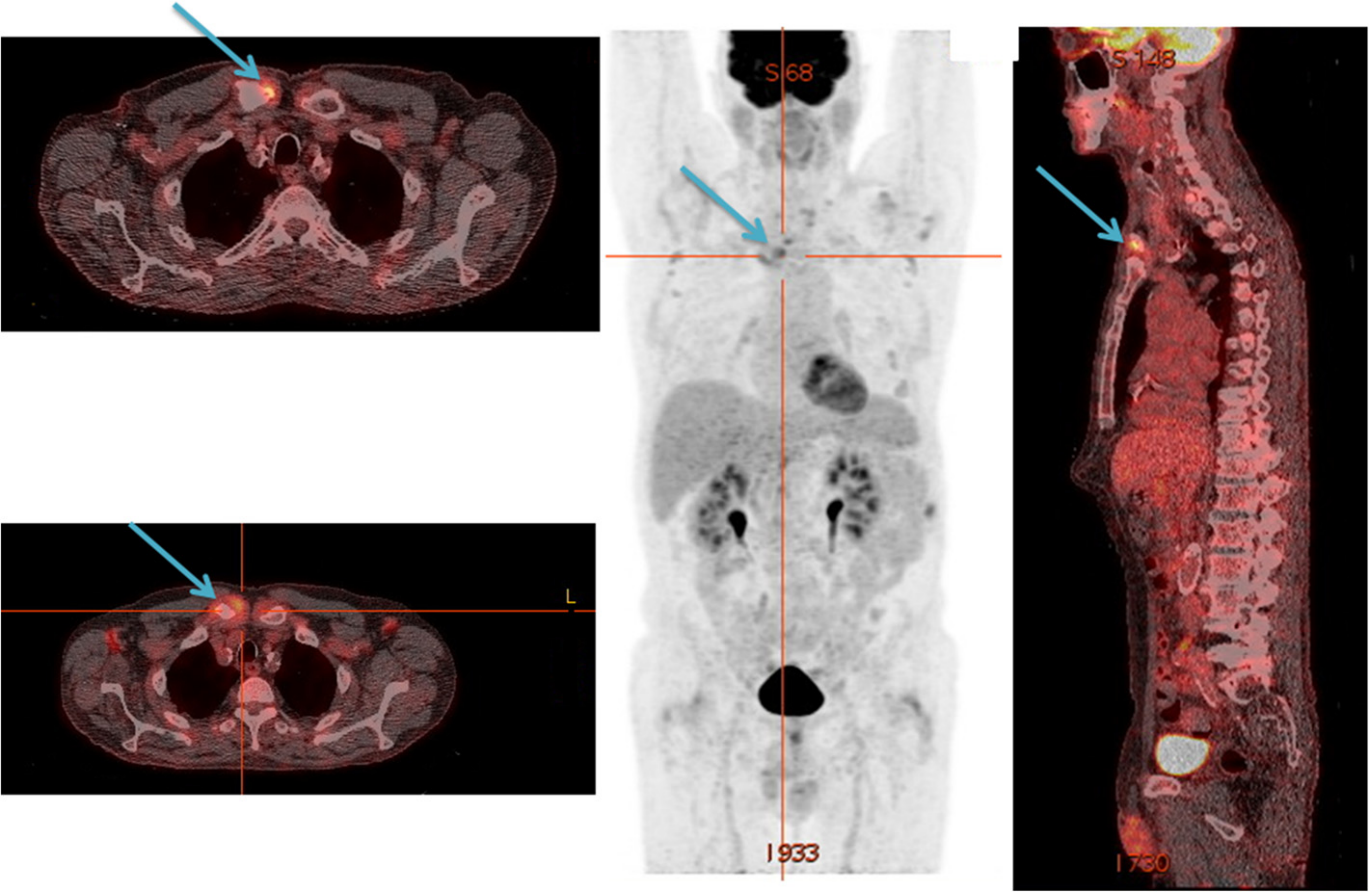
^18^F-fluorodeoxyglucose positron emission tomography computed tomography in a patient with *Coxiella burnetii* sternoclavicular joint infection. The high fluorodeoxyglucose uptake (*arrows*) is observed at the level of the sternoclavicular joint

A 67-year-old French man from south France was admitted to our hospital approximately 1 year ago (February 2015) with fever (39 °C) and confusion. He mentioned increased alcohol consumption. An examination revealed subclavicular and axillary lymph node enlargement. Moreover, he presented cerebellar ataxia, with loss of equilibrium and difficulty walking. Laboratory values revealed elevated C-reactive protein (216 mg/L) and liver enzyme levels (aspartate aminotransferase 100 IU/L, alanine aminotransferase 69 IU/L, gamma-glutamyl transferase 125 IU/L) and hyponatremia (sodium 123 mEq/L). A liver ultrasound showed hepatomegaly. Computed tomography (CT) scanning was normal and magnetic resonance imaging (MRI) scanning did not reveal signs of infection. Treatment with ceftriaxone and levofloxacin was introduced, but his fever did not resolve. A serum sample was sent to our laboratory in Marseille and an immunofluorescence assay (IFA) was positive for *C. burnetii*; phase I titers for immunoglobulin (Ig) G, IgM, and IgA were 400, 200 and 0, respectively, and phase II titers were 400, 200, and 0, respectively. The diagnosis of acute Q fever infection was made and he was treated with 200 mg oral doxycycline daily for 21 days. IgG anticardiolipin (aCL) antibody levels in the serum sample were very high (216 GPLU), and we suspected valvular heart disease and a possible progression to Q fever endocarditis [5]. However, a transesophageal echocardiogram was normal. After 2 months we obtained a second serum sample and phase I IFA titers for IgG, IgM, and IgA were 25,600, 0 and 0, respectively, and phase II titers were 25,600, 0, and 0, respectively. Both serum samples were negative for *C. burnetii* by quantitative polymerase chain reaction (qPCR) for the IS1111 and the IS30A spacers [9]. A localized *C. burnetii* infection was suspected; lymph node biopsies were performed that were negative for *C. burnetii* by molecular assays. For each sample, we verified the quality of DNA handling and extraction of samples by qPCR for a housekeeping gene encoding beta-actin [10]. The lymph node biopsies were also negative for *C. burnetii* by immunohistochemical analysis using a monoclonal antibody against *C. burnetii* with an immunoperoxidase kit [11]. Moreover, the lymph nodes were also tested by fluorescent *in situ* hybridization (FISH) [12], which was also negative. To localize the site of the infection we performed PET scanning, which revealed intense fluorodeoxyglucose uptake in his right sternoclavicular joint (Fig. 1). A diagnosis of sternoclavicular joint infection by *C. burnetii* was made, and treatment with 200 mg oral doxycycline daily and 200 mg oral hydroxychloroquine three times daily for 18 months was introduced. After 6 months follow-up, the outcome was favorable, with a four-fold decrease in the phase I and phase II IFA titers for IgG.

## Discussion

We report a case of Q fever sternoclavicular joint infection diagnosed through serology and localized using PET scanning. PET scanning has been previously used for the identification of infectious foci in *C. burnetii* vascular infections [7], in the bone marrow [13], in the liver [14], and recently two cases of arthritis and subacromial bursitis caused by *C. burnetii* were also localized [1]. In this case we suspected a localized *C. burnetii* infection because of the very high IFA titers and IgG-aCL levels [15]. In fact, persistent localized infections have been associated with increased levels of IgG and IgA antibodies [16]. However, echocardiography did not reveal signs of endocarditis and lymph nodes were negative for *C. burnetii.* As Q fever articular infections present a long evolution of articular involvement, accompanied by a low level of inflammatory signs, and can easily remain undiagnosed [2], the PET scanner was a valuable tool for the identification and the localization of the infectious foci of *C. burnetii* in the sternoclavicular joint. Although we did not test the sternoclavicular joint to confirm the diagnosis, a localized infection with *C. burnetii* is associated with high antibody titers against *C. burnetii* [17]. For our patient the only fluorodeoxyglucose uptake was in the right sternoclavicular joint indicating that this was the site of fixation.

Epidemiological patterns of Q fever osteoarticular infections may consist of sporadic cases that are difficult to diagnose. As with the majority of Q fever osteoarticular infections, our patient was an adult man more than 50-years old [2]. Although most described cases with osteoarticular infections present swelling and arthralgia without inflammatory signs [2, 6], our patient did not complain of arthralgia. Despite limited data concerning therapeutic strategies for Q fever osteoarticular infections, a regimen of doxycycline and hydroxychloroquine for 18 months has been suggested [1, 2]. Our patient was treated with a course of doxycycline and hydroxychloroquine for 18 months.

## Conclusion

In conclusion, we confirm the efficacy of PET scanning for the identification of infectious foci in *C. burnetii* infections and we strongly urge its use in patients with high *C. burnetii* antibody titers in order to localize the site of the *C. burnetii* infection.

## Abbreviations

aCL, anticardiolipin; CT, computed tomography; FISH, fluorescent *in situ* hybridization; IFA, immunofluorescence assay; Ig, immunoglobulin; MRI, magnetic resonance imaging; PET, positron emission tomography; qPCR, quantitative polymerase chain reaction.
